# Modulating antibody N-glycosylation through feed additives using a multi-tiered approach

**DOI:** 10.3389/fbioe.2024.1448925

**Published:** 2024-08-26

**Authors:** Jaka Kranjc, Lovro Kramer, Miha Mikelj, Marko Anderluh, Anja Pišlar, Matjaž Brinc

**Affiliations:** ^1^ Institute of Pharmacy, Faculty of Pharmacy, University of Ljubljana, Ljubljana, Slovenia; ^2^ Cell Line Engineering and Characterization, Technical Research & Development, Novartis Pharmaceutical Manufacturing LLC, Mengeš, Slovenia; ^3^ Process Analytical Science, Technical Research & Development, Novartis Pharmaceutical Manufacturing LLC, Mengeš, Slovenia; ^4^ Department of Pharmaceutical Chemistry, Faculty of Pharmacy, University of Ljubljana, Ljubljana, Slovenia; ^5^ Department of Pharmaceutical Biology, Faculty of Pharmacy, University of Ljubljana, Ljubljana, Slovenia; ^6^ Process Development, Technical Research & Development, Novartis Pharmaceutical Manufacturing LLC, Mengeš, Slovenia

**Keywords:** bioprocess, glycosylation, modulators, modelling, Chinese hamster ovary, antibody production

## Abstract

Glycosylation of recombinant proteins is a post-translational modification that affects multiple physicochemical and biological properties of proteins. As such, it is a critical quality attribute that must be carefully controlled during protein production in the pharmaceutical industry. Glycosylation can be modulated by various conditions, including the composition of production media and feeds. In this study, the N-glycosylation-modulating effects of numerous compounds, including metal enzyme cofactors, enzyme inhibitors, and metabolic intermediates, were evaluated. Chinese hamster ovary cells producing three different IgG antibodies were cultivated in a fed-batch mode. First, a one-factor-at-a-time experiment was performed in 24-well deep well plates to identify the strongest modulators and appropriate concentration ranges. Then, a full response surface experiment was designed to gauge the effects and interactions of the 14 most effective hit compounds in an Ambr^®^ 15 bioreactor system. A wide range of glycoform content was achieved, with an up to eight-fold increase in individual glycoforms compared to controls. The resulting model can be used to determine modulator combinations that will yield desired glycoforms in the final product.

## 1 Introduction

Protein glycosylation, i.e., the enzymatic attachment of carbohydrate moieties to proteins, plays a central role in numerous biological processes, including cell recognition, immune responses, and protein stability (Cymer et al., 2018). The complexity of glycan structures contributes to the structural diversity of glycoproteins (Moremen et al., 2012). Glycosylation strongly affects protein properties, and most therapeutic proteins are glycosylated. Thus, glycosylation is often considered a critical quality attribute during the production of biopharmaceuticals (Edwards et al., 2022). Modulating glycosylation enables the generation of different glycosylation variants, including high-mannose (HM), afucosylated, galactosylated, and sialylated glycoforms. The most effective and direct control of glycosylation can be achieved by genetically engineering the production cell line. Altering the expression level of specific enzymes in the glycosylation pathway shifts the glycoform ensemble towards the desired composition (Tejwani et al., 2018). This process, however, is very complex and time-consuming. Thus, using external factors to alter glycosylation profiles is becoming a commonly used method. The simplest and most researched factors are related to cell cultivation, such as the proportion of dissolved gasses (Nguyen Dang et al., 2019), temperature (Borys et al., 2010), pH (Nguyen Dang et al., 2019), and osmolality (Alhuthali et al., 2021). The drawback of these methods is that they have a broad effect on cell metabolism, which prevents specific regulation of individual glycoforms. Furthermore, thorough experimentation is required to establish optimal parameter ranges across different cell lines.

These problems can be circumvented by adding glycosylation modulators that affect glycosylation profiles. Precise control over glycosylated species can be achieved by using inhibitors that target a specific enzyme or enzyme family in the glycosylation pathway (Yu et al., 2011). Using precursors of different molecules of the glycosylation pathway has been reported to be effective in increasing enzymatic activity (Kildegaard et al., 2016). Additionally, changing the concentration of metal ions, which function as enzyme cofactors, can effectively alter glycosylation (Markert et al., 2020). Chemical modulators also enable more precise control of glycosylation species by adjusting their concentrations and feeding regimens (exposure times) and by concurrently using more than one modulator (Ehret et al., 2018).

In the present work, the effects of numerous glycosylation modulators were systematically investigated in a multi-tiered experiment to find the most effective candidates that could be used in process development, and their impacts and interactions were characterized. An initial screening of 38 modulators was performed in 24-well deep well plates (DWPs) to determine the maximum non-toxic effective concentrations. For this experiment Chinese hamster ovary (CHO) cell line derived from a parental CHO-K1 line producing an IgG molecule (IgG A) was used. Based on the findings of screening, factors for a design of experiment (DoE) response surface experiment were chosen. In the following, two available CHO cell lines derived from a parental CHO-K1 line producing different IgG molecules (IgG B and IgG C) were cultivated for the response surface experiment to compare the consistency of modulator effects across different cell lines. The DoE experiment was performed in an Ambr^®^ 15 bioreactor system under controlled process conditions and included 165 bioreactors. Afterwards, a DoE model describing the effect of the investigated modulators on each glycan group and on antibody titer was constructed. Additionally, all first- and second-order interactions and quadratic effects were successfully elucidated by the model. With the use of this model, it is possible to determine the optimal concentrations and combinations of modulators required to achieve the desired glycosylation profile. Although additional validation experiments are required for the method’s use in production systems, the data presented in this paper serves as an effective guide for selecting glycosylation modulators.

## 2 Materials and methods

### 2.1 Cell cultivation and bioprocess conditions

Three different CHO cell lines derived from an internally developed CHO-K1 line producing different IgG molecules (IgG A, IgG B, and IgG C) were used. After thawing, the cells were cultivated in shake flasks (Corning, cat. Nr.: #431143, #431144, #431145) in proprietary chemically defined media provided by Irvine Scientific and passaged at least four times to enable sufficient recovery from thawing. Shake flask cultures were cultivated in ISF1-X incubator shakers (Kuhner shaker) at 36.5°C and 10% CO_2_, with a shaking rate of 200 rpm and radius of 12.5 mm.

Cells were cultivated in three different setups. Cells cultivated in shake flasks were used as inoculum, whereas cells cultivated in 24-well DWPs and Ambr^®^ 15 bioreactors were used to test glycosylation modulators. Cells were cultivated according to an in-house proprietary fed-batch platform.

The first round of modulator screening was performed in 24-well DWPs with a working volume of 3 mL/well. For this stage of the experiment, IgG-A-producing cells were cultivated in incubators at 36.5°C, 10% CO_2_, and 80% humidity, with a shaking rate of 300 rpm and radius of 25 mm. Cells were cultivated in a proprietary protein-free chemically defined medium. All wells were seeded with the same inoculum to a target density of 0.4 × 10^6^ viable cells/mL and were cultivated for 14 days. On day 4, the temperature was decreased to 33 °C, and feeding with glucose was started. The first feed with glucose and amino acids was added on days 4, 6, 7, 10, and 12. The second feed with additional amino acids was added on days 7, 10, and 12. On days 7, 10, and 14, wells were sampled for cell density measurements with Cellavista (SYNENTEC GmbH). On day 14, product titer was measured by bio-layer interferometry (Octet, Sartorius). Glycosylation modulators were added to cell cultures according to the feeding plan and modulator type (Table 1). Enzyme inhibitors and metal cofactors were added in a bolus feed on day 7, and metabolic precursor modulators were added in three bolus feeds on days 7, 10, and 12. Selected sugars were also added as part of the glucose feed. Apart from the modulators included in the glucose feed, all feeds had the same volume of 30 μL. Modulators soluble in water were dissolved in water for injection, whereas modulators soluble in organic solvents were dissolved in dimethyl sulfoxide (DMSO). Before use, concentrated DMSO solutions were diluted (1:1) with water for injection, and thus the final DMSO concentration in cell cultures never exceeded 0.5% (v/v). Table 1 displays a list of the modulators used in this stage of the experiment as well as the expected effect, feeding regimen, highest tested concentration, and dilution steps. All chemicals were supplied by Sigma-Aldrich. On day 14, after sampling for cell density, all 24-well DWPs were centrifuged at 3000 G for 10 min (5810 R centrifuge, Eppendorf, cat. Nr: #5811000015). After centrifugation was complete, the supernatant from each well was transferred to 96-well DWPs and frozen at −80°C. N-glycan profiles of selected samples were then analyzed as described in Section 2.3.

**TABLE 1:** List of all modulators used in the deep well plate screening.

Substance name	Reported N-glycan modulation	Proposed mechanism of action	Feeding and dilution regimen	Highest tested concentration	Reference
Kifunensine	HM ↑	Enzyme inhibitor	A	43 μM	Ehret et al. (2018)
Siastatin B	SIA ↑	Enzyme inhibitor	A	100 μM	Li and McClane (2014)
Sodium butyrate	GAL ↑	Enzyme inhibitor	A	9 mM	Hong et al. (2014)
Monensin*	HM ↑	Ionophore	A	997.8 nM	Pande et al. (2015)
Dexamethasone*	SIA ↑	Altered gene expression	A	9999.31 nM	Jing et al. (2010)
NGI-1*	Aglycosylation ↑	Enzyme inhibitor	A	100 μM	Rinis et al. (2018)
GDP-2F-fucose	AFUC ↑	Enzyme inhibitor	A	790 μM	Rillahan et al. (2012)
2,3-Dehydro-2-deoxy-N-acetylneuraminic acid	SIA ↑	Enzyme inhibitor	B	1670 μM	Gramer et al. (1995)
1-Deoxynojirimycin hydrochloride	HM ↑	Enzyme inhibitor	A	1000 μM	Elbein (1991)
Nojirimycin bisulfite	HM ↑	Enzyme inhibitor	A	500 μM	Kodama et al. (1985)
Methylenediphosphonic acid	GAL ↓	Enzyme inhibitor	A	1000 μM	Wang and Vidal (2013)
Deoxygalactonojirimycin hydrochloride	HM ↑	Enzyme inhibitor	A	0.1 μM	Wang and Vidal (2013)
N-butyldeoxynojirimycin	HM ↑	Enzyme inhibitor	A	10 μM	Platt et al. (1994)
MnCl_2_	GAL ↑	Metal cofactor	A	126 μM	Gramer et al. (2011)
NH_4_Cl	GAL ↓	Toxic by-product	A	20 mM	St. Amand et al. (2014)
Copper (II) sulfate	SIA ↑	Metal cofactor	A	80 μM	Mitchelson et al. (2017)
ZnCl_2_	GAL ↓	Metal cofactor	A	800 μM	Prabhu et al. (2018)
CoCl_2_	GAL ↑	Metal cofactor	A	80 μM	Hossler and Racicot (2015)
Iron (III) chloride	Aglycosylation ↓	Metal cofactor	A	3.37 mM	Gawlitzek et al. (2009)
Lithium chloride	SIA ↓	Metal cofactor	A	10 mM	Ha et al. (2014)
Sucrose	HM ↑	Sugar substrate	B	175 mM	Ehret et al. (2018)
Mannose	HM ↑	Sugar substrate	B	200 mM	Ehret et al. (2018)
Tagatose	HM ↑	Sugar substrate	B	16 mM	Ehret et al. (2018)
Raffinose	HM ↑	Sugar substrate	B	14 mM	Brühlmann et al. (2017)
Galactose	GAL ↑	Sugar substrate	B	80 mM	St. Amand et al. (2014)
N-Acetylmannosamine	SIA ↑	Other substrate	B	17 mM	Wong et al. (2010)
Glucosamine	SIA ↑	Other substrate	B	14 mM	Wong et al. (2010)
UDP-galactose	GAL ↑	Sugar substrate	B	1000 μM	Sumit et al. (2019)
Lactose	GAL ↑	Sugar substrate	B	15 mM	Hossler et al. (2017)
Lactulose	GAL ↑	Sugar substrate	B	66.69 mM	Hossler et al. (2017)
Asparagine	GAL ↓	Other substrate	B	6.68 mM	McCracken et al. (2014)
N-Acetylglucosamine	GAL ↓	Other substrate	B	6.78 mM	Kildegaard et al. (2016)
D-arabinose	AFUC ↑	Sugar substrate	B	10 mM	Hossler et al. (2017)
L- fucose	AFUC ↓	Sugar substrate	B	9.14 mM	Kildegaard et al. (2016)
N-acetylneuraminic acid	SIA ↑	Other substrate	B	4.85 mM	Savinova et al. (2015)
Cytidine	SIA ↑	Other substrate	B	12.34 mM	Wong et al. (2010)
Uridine	GAL ↑	Other substrate	B	12.28 mM	Kildegaard et al. (2016)
Ex-Cell GAL+	GAL ↑	Other substrate	B	3% (v/v)	Anderson et al. (2018)
Sucrose	HM ↑	Sugar substrate	C	66.6% of total sugars	Ehret et al. (2018)
Mannose	HM ↑	Sugar substrate	C	100% of total sugars	Ehret et al. (2018)
Tagatose	HM ↑	Sugar substrate	C	50% of total sugars	Ehret et al. (2018)
Galactose	GAL ↑	Sugar substrate	C	83.3% of total sugars	St. Amand et al. (2014)
Lactose	GAL ↑	Sugar substrate	C	66.6% of total sugars	Hossler et al. (2017)
L-fucose	AFUC ↓	Sugar substrate	C	66.6% of total sugars	Kildegaard et al. (2016)

* denotes solutions prepared using DMSO. All other solutions were prepared using water for injection. Feeding and dilution regimens were as follows: A) four serial log dilutions of stock solution, each fed on day 7; B) four serial 1:2 dilutions of stock solution, each fed on days 7, 10, and 12; C) four serial 1:2 dilutions of stock solution, each fed as part of the glucose feed. AFUC, afucosylated; GAL, galactosylated; HM, high-mannose; SIA, sialylated.

The DoE study was performed in an Ambr^®^ 15 (Sartorius) bioreactor system with 48 culture vessels. In total, four consecutive experimental campaigns were carried out to obtain the bioreactor runs reported in this study. Two different cell lines that produce either IgG B or IgG C were used. The seeding density was 0.4 × 10^6^ viable cells/mL, and the initial working volume of one microbioreactor was 14.5 mL. Cells were cultivated for 14 days, with a starting temperature of 36.5°C, which was decreased to 33°C after the average cell density in a culture station reached a predetermined set point. The pH set point was 6.95 with a dead-band of 0.1. Culture pH was controlled by CO_2_ sparging and by adding 0.5 M NaOH solution on demand. The agitation rate was 700 rpm counterclockwise. Glucose and amino acid feeding and additional amino acid feeding was started on day 3 and was applied daily until including day 13. Glycosylation modulators were added in three regimens: A) in one bolus feed on day 7; B) on days 7, 9, and 11; and C) daily as part of the glucose/sugar feed from day 7 onwards. The modulators used in this experiment were kifunensine, MnCl_2_, monensin, dexamethasone, mannose, galactose, lactose, L-fucose, N-acetylglucosamine, uridine, Ex-Cell GAL+, 1-deoxynojirimycin, N-acetylmannosamine (all from Sigma-Aldrich), and 2F-peracetyl fucose (Carbosynth). On days 0, 3, 5, 7, 10, 12, and 14, bioreactors were sampled for measurements of cell density (Vi-CELL XR, Beckman Coulter) and metabolite and antibody titer (Cedex Bio HT, Roche) (data not shown). On day 14, all bioreactors were harvested by transferring their contents to centrifuge tubes (Corning, cat. Nr: #430828), which were centrifuged at 3200 G for 10 min (5810 R centrifuge, Eppendorf, cat. Nr: #5811000015). After centrifugation, the supernatant was filtered through 0.45 μm Steriflip tubes (Millipore, #SE1M003M00), aliquoted, and frozen. Samples from all bioreactors were then analyzed for their antibody titer (Section 2.2) and N-glycan profile (Section 2.3).

### 2.2 Antibody titer determination

The titer of produced recombinant IgG antibodies was determined by protein A chromatography. High-performance liquid chromatography was performed with the Agilent LC 1200 quaternary system with an autosampler and variable injection volume (5–50 μL). The column used was a Poros PA ImmunoDetection Sensor Cartridge for Perfusion Immunoassay (Protein A), 2.1 mm × 30 mm, 20 μm (Applied Biosystems, cat. Nr.: #2-1001-00). The assay was run according to the column manufacturer’s protocol (Thermo Scientific, 2018). The loading buffer contained 10 mM NaH_2_PO_4_, 150 mM NaCl, pH 7.5, and the elution buffer contained 10 mM HCl, 150 mM NaCl, pH 2.0. The flow rate was 2 mL/min, and the detection wavelength was 280 nm. The chromatography peaks were integrated with Empower Chromatography Data Software (Waters).

### 2.3 N-glycan analysis

Samples were prepared for N-glycan analysis using a commercially available kit produced by Agilent. The kit contained an Agilent AdvanceBio Gly-X deglycosylation module, InstantPC labeling module, and InstantPC, which were used following the manufacturer’s protocol (Agilent Technologies, 2023).

N-glycans were labelled and analyzed by hydrophilic interaction chromatography using a fluorescence detector. An ACQUITY UPLC H-Class system (Waters) with a FLR detection module coupled with an ACQUITY UPLC BEH Glycan 130 Å, 1.7 µm 50 × 2.1 mm column (Waters) was used. The injection volume was 1 μL/sample, and the temperatures of the autosampler and column were set to 5°C and 60°C, respectively. For chromatography, two mobile phases with a variable gradient were used, as recommended by Agilent. Mobile phase A contained 100% acetonitrile (Honeywell, cat. Nr.: #34967), and mobile phase B contained 50 mM ammonium formate solution (Waters, cat. Nr.: # 186007081). The duration was 33 min/sample, and the obtained peaks were integrated with Empower software (Waters).

### 2.4 Design of experiments and statistical evaluation

For the first screening round in 24-well DWPs, a wide concentration range for each compound was tested. Either four log dilutions or four two-fold serial dilutions were used, depending on the compound (Table 1). In this way, the maximum non-toxic concentrations at which the compounds exhibited maximum efficacy were determined.

An experimental design using JMP 14.2 software (SAS) was prepared for the Ambr^®^ 15 bioreactor system experiment. An I-optimal response surface design with 14 continuous factors (glycosylation modulators), one two level categorical factor (two different IgG molecules: IgG B and IgG C), and a blocking factor (one block corresponds to 12 bioreactors, i.e., one Ambr^®^ 15 culture station) were created. A linear constraint limiting the sum of mannose, lactose, galactose, and L-fucose to <85.7% of total fed glucose in the standard platform process feed was used. In this way, the cumulative sugar feed was not allowed to exceed the maximum feed rate of the platform process. The created design contained 168 runs in 14 blocks and was fully completed in four Ambr^®^ 15 campaigns. The design table with factor levels for all runs can be found in Supplementary Table S1. During the experiment, three bioreactors were lost due to contamination, decreasing the number of bioreactors included in the final model to 165. The rows containing these experimental runs were removed from the design table in Supplementary Table S1.

After all data was obtained, a multiple-linear regression model comprising the main effects, all two-factor interactions, and all quadratic effects was fitted in JMP 17 software (SAS). Statistically insignificant model terms were removed.

## 3 Results and discussion

### 3.1 Screening in 24-well DWPs

**FIGURE 1:**
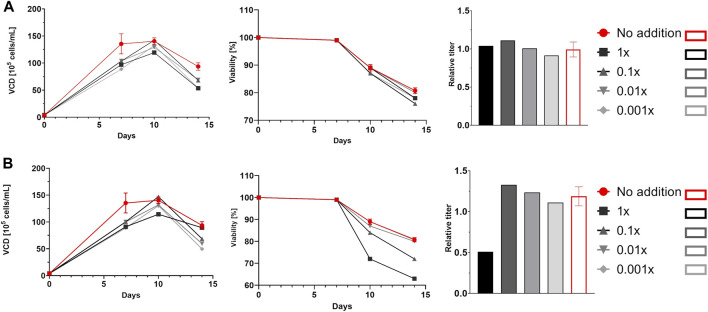
Viable cell density (VCD) profiles, viability profiles, and relative titers. Cells cultured in deep well plates were supplemented with siastatin B **(A)** or ZnCl_2_ **(B)**. Red lines and bars represent controls, and different shades of grey represent different concentrations of siastatin B and ZnCl_2_. For controls, error bars representing the standard deviation of the mean of three parallel runs are shown.

**FIGURE 2:**
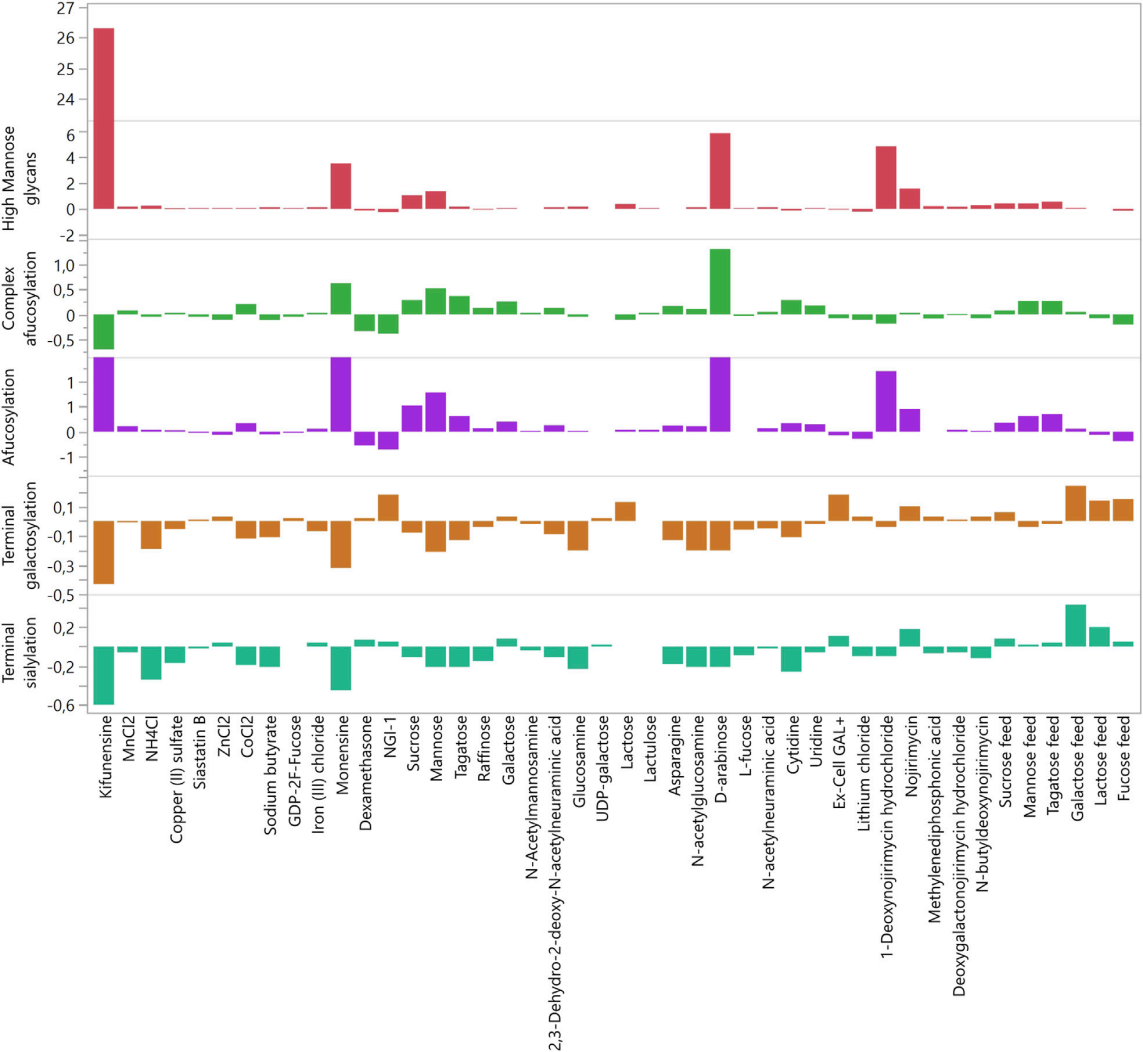
Fold changes (relative to control) of glycan groups caused by modulators in the deep well plate experiment. The highest non-toxic concentration of each modulator was used.

Cells were cultured according to an in-house standardized platform process and behaved as expected, with homogenous growth profiles up to day 7 (data not shown). Some differences in viability and product titer were observed after the addition of glycosylation modulators, especially at the highest concentrations. For example, ZnCl_2_ decreased both viability and productivity at the highest concentration, whereas siastatin B did not exert any significant effects on growth or productivity across the entire concentration range (Figure 1). The results of the glycan analysis are shown in Figure 2. The glycan species abundances were normalized versus the appropriate control, and thus the values displayed are fold changes in glycan species abundance. In some cases, the observed effects on glycosylation were consistent with those described in the literature, whereas in other cases, the effects were absent or even opposite to those reported.

The measured glycan structures were divided into five groups: HM, afucosylated, complex afucosylated, galactosylated, and sialylated. The results of glycosylation analysis for each modulator used in the DWP experiment are presented in Figure 2. The exact modulator concentrations and fold changes in glycosylation can be found in Supplementary Table S2. The strongest effects were observed in the HM group by mannosidase inhibitors such as kifunensine and 1-deoxynojirimycin, which increased the proportion of HM glycans by up to 26-fold (Figure 2). Additionally, D-arabinose increased HM glycans by almost six-fold, which is in accordance with previously published data (Hossler et al., 2017). However, the effects of D-arabinose appear to be very cell line-specific. Hossler et al. reported that D-arabinose decreased HM glycan abundancy in two cell lines but increased HM glycan abundancy in a third line. In the present study, 10 mM D-arabinose increased HM glycans by six-fold. Conversely, the abundancy and efficacy of modulators that decreased HM glycans was much lower. The strongest effect was shown by NGI-1 (24%) followed by cytidine (13%), dexamethasone (12%), and copper (6%).

HM glycans lack core fucose and are thus afucosylated. Hence, a similar trend was observed in the afucosylation group, in which the strongest modulators were the ones mentioned above. To determine the modulators that affect afucosylation independently of the increase in HM glycans, HM glycan content values were subtracted from the afucosylation values, thus creating the complex afucosylation group. This was done before the data was normalized. Comparing the values of complex afucosylation versus afucosylation revealed modulators that non-specifically affect afucosylation (Figure 2). The most glaring example is kifunensine, which increased afucosylation by 7.3-fold yet decreased complex afucosylation of the targeted antibody by 71%. Monensin also affected afucosylation, mainly through the increase in HM glycans, but even after accounting for HM glycans it still increased complex afucosylation by 63%. The most promising modulator of afucosylation was D-arabinose as it increased complex afucosylation by 1.32-fold even when subtracting the increase in HM glycans. However, unknown glycan chromatographic peaks were present in the samples in which D-arabinose was used. Upon analysis with mass spectrometry (data not shown), the unknown peaks were confirmed to be glycans with incorporated D-arabinose instead of L-fucose, which is why the afucosylation level was increased. These findings are in accordance with previous reports (Hossler et al., 2017). As replacement of glycan components with alternate sugars was not within the scope of this study, D-arabinose was eliminated as a potential glycomodulator.

Interestingly, L-fucose did not decrease complex afucosylation when added as a bolus feed. The suspected cause of this is the relatively low concentration of L-fucose compared to glucose in the cell growth media. Therefore, the approach was changed in the second pre-screening round, and L-fucose was fed as part of the glucose feed, replacing up to 66.6% of total glucose feed. In this configuration, L-fucose decreased complex afucosylation by 27% but also significantly decreased product titer. Thus, 33.3% of L-fucose was also analyzed to determine whether the use of a lower concentration is feasible. Glucose feed with 33.3% of L-fucose decreased complex afucosylation by 14%, with no negative impact on titer. In addition, L-fucose decreased the proportion of HM glycans and increased galactosylation at both concentration levels.

In the first round of experiments, the largest increase in galactosylation was achieved by NGI-1 and Ex-Cell GAL+ (18%), followed by lactose (13%) (Figure 2). Galactose fed as a bolus feed marginally increased galactosylation. This was very similar to the previously described lack of effect with L-fucose, and thus galactose was also fed as a glucose replacement in the second experiment. Replacing 83.3% of fed glucose with galactose increased galactosylation by 24%. Lactose fed as glucose replacement also had a beneficial effect on galactosylation and sialylation, increasing galactosylation by 14%. Decreases in galactosylation were mainly caused by modulators that increased HM glycan species. Interesting results were found for cobalt which is reported to increase the abundance of galactosylated species (Hossler and Racicot, 2015) yet decreased galactosylation by up to 20% in this study.

Terminal sialylation heavily depends on both HM and galactosylated glycoforms as it is the most “mature” glycan group. Thus, it is not surprising that the same modulators that increased the abundance of HM species strongly decreased the abundance of sialylated glycoforms. The most prominent examples of such modulators are kifunensine and monensin, followed by NH_4_Cl and sodium butyrate (Figure 2). Interestingly, the relationship between the increase in HM species and decrease in sialylated species does not seem to be linear. This is most noticeable when comparing kifunensine (which increased HM species by 26-fold and decreased sialylated species by 60%) with NH_4_Cl (which increased HM species by 25% and decreased sialylated species by 34%).

Similarly, sialylation was most increased by modulators that also increased galactosylation. The most prominent effect was caused by galactose (43%) and lactose (20%) when both were fed as partial glucose replacements (Figure 2). Ex-Cell GAL+, which increased galactosylation, also increased sialylation, but to a smaller extent (11%). An interesting effect was exerted by nojirimycin, a β-glucosidase inhibitor. This modulator increased the abundance of both HM and sialylated glycoforms relatively strongly (sialylation increased by 20%). In increasing sialylated glycoforms, it even outperformed Ex-Cell GAL+, which was not expected. Another surprising finding was related to 2,3-dehydro-2-deoxy-*N*-acetylneuraminic acid. This molecule is an inhibitor of sialidases, i.e., extracellular enzymes that remove exposed sialic acid residues from glycans (Keil et al., 2022). As such, the expected effect was an increase in sialylated species, as the removal of sialic acids by extracellular sialidases should be inhibited. Contrary to expectations, 2,3-dehydro-2-deoxy-*N*-acetylneuraminic acid decreased the abundance of sialylated glycoforms by 11%, in contrast to previous findings (Gramer et al., 1995). Another modulator that did not exhibit expected effects was *N*-acetylneuraminic acid, a precursor from which cells synthesize sialic acids. In our study, this precursor decreased sialylation by 2%, rather than increasing sialyation as previously reported (Savinova et al., 2015). Similarly, although cytidine has been reported to increase sialylation (Wong et al., 2010), in our study, it decreased sialylation by 26%.

### 3.2 Response surface DoE

**FIGURE 3:**
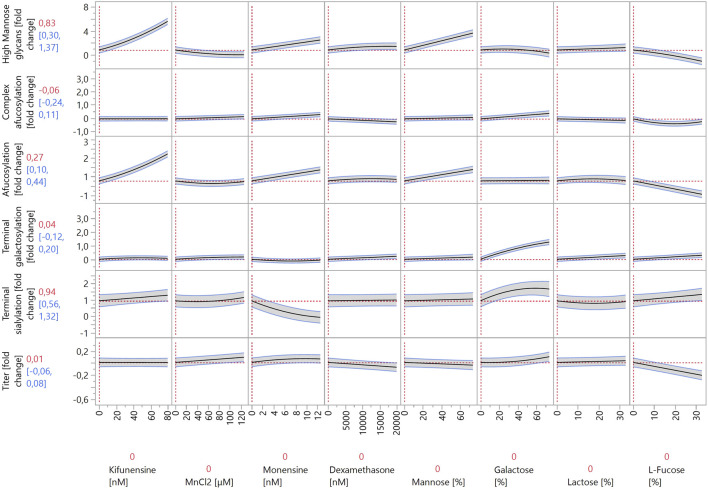
Prediction profiler created by JMP 17 software. The prediction profiler displays predicted responses (y-axes) to changes in variables (x-axes). The x-axes show the concentrations of the modulators included in the model, whereas the y-axes show the fold changes in glycosylation species relative to control experiments. The vertical dotted lines indicate the current value or setting for each variable/modulator on the x-axes. The horizontal dotted lines indicate the predicted values of the y-axis variables for the current settings of all x-axis variables. The shaded areas around the solid black lines show the 95% confidence interval for the predicted values of continuous factors.

**FIGURE 4:**
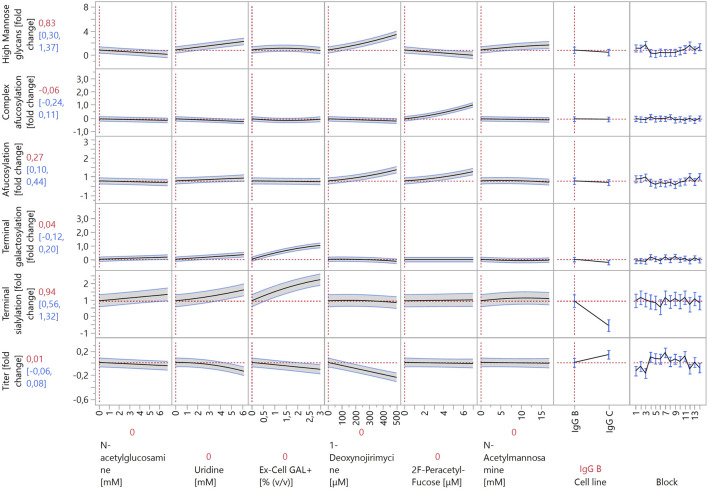
Prediction profiler created by JMP 17 software. The prediction profiler displays predicted responses (y-axes) to changes in variables (x-axes). The x-axes show the concentrations of the modulators included in the model (or the settings of categorical factors), whereas the y-axes show the fold changes in glycosylation species relative to control experiments. The vertical dotted lines indicate the current value or setting for each variable/modulator on the x-axes. The horizontal dotted lines indicate the predicted values of the y-axis variables for the current settings of all x-axis variables. The shaded areas around the solid black lines show the 95% confidence interval for the predicted values of continuous factors. For categorical factors, the 95% confidence interval is shown as error bars.

Cells producing IgG B and IgG C were grown in Ambr^®^ 15 as part of the response surface DoE. Glycosylation modulator concentrations were chosen based on the screening results (Figure 2). Both cell lines were cultivated according to the same platform process and had very similar growth curves and metabolite profiles up to day 7 (data not shown). More variability in growth and cell metabolism was present due to different feeds and modulators as specified by the experimental design (data not shown). The JMP prediction profiler for the models created based on the experimental results is shown in Figures 3, 4. The full report including model effect summaries, parameter estimates, and interaction plots can be found in html format in Supplementary Table S3. The submitted glycan data has been normalized using two separate control runs, one for each IgG molecule. Thus, the y-axes in the JMP prediction profiler represent fold changes of the respective glycan structures or titer. The quality of the constructed models is not affected by the data obfuscation used.

All six models have high R^2^ values with no overfitting of data. Several diagnostics were performed using JMP 17 to confirm that no overfitting is present (Supplementary Table S3). The high strength of the created models was expected, as only modulators with (relatively) strong effects were used in this experiment. A summary of the identified modulator effects is shown in Table 2 below.

**TABLE 2:** Summarized effects of modulators on investigated glycan groups and product titer as identified by the constructed models.

Modulator	HM	Complex AFUC	AFUC	Terminal GAL	Terminal SIA	Titer
Kifunensine	+++	0	+++	0	+	0
MnCl2	--	+	0	+	+	++
Monensine	++	+	++	-	---	+
Dexamethasone	+	--	0	+	0	-
Mannose	+++	+	++	+	+	-
Galactose	-	++	0	+++	+++	++
Lactose	+	-	0	++	0	0
L-Fucose	---	---	---	++	++	---
N-acetylglucosamine	--	-	0	+	++	-
Uridine	++	-	+	++	++	--
Ex-Cell GAL+	0	0	0	+++	+++	-
1-Deoxynojirimycine	+++	-	++	--	0	---
2F-Peracetyl-Fucose	--	+++	++	0	0	0
N-Acetylmannosamine	+	0	0	-	+	0

+++ denotes very strong increase, --- denotes very strong decrease, and 0 denotes no significant effect on the specified glycan group.

When comparing the increases in HM species in Ambr^®^ 15 versus 24-well DWPs, the same modulators exerted the largest effects: kifunensine, monensin, 1-deoxynojirimycin, and mannose. As the concentrations of the first three listed modulators were significantly decreased for the Ambr^®^ 15 experiment, it is not possible to directly compare their effects to those of the 24-well DWP experiment. Concentrations were decreased to prevent very high increases in HM species from negating the effects of other modulators. Conversely, mannose was used at a similar concentration, and, surprisingly, it exhibited a much more pronounced effect in the Ambr^®^ 15 system. High mannose concentrations increased the abundance of HM species by almost three-fold in the Ambr^®^ 15 system but exerted only an 43% increase in 24-well DWPs. It is possible that the cell lines used in the Ambr^®^ 15 experiment utilize mannose more effectively in their metabolism than the one used in the DWPs and can incorporate mannose into glycoproteins at higher rates. Another possibility is that pH plays a role. The pH levels in 24-well DWPs were not controlled, leading to lower cell media pH and significantly altered cell metabolism (Jiang et al., 2018). The same reasons probably underlie the increased effects of galactose, L-fucose, and N-acetylglucosamine on decreasing HM species. In 24-well DWPs, these modulators exhibited a very weak (or zero) effect on HM glycoforms. Under DoE conditions, both galactose and N-acetylglucosamine decreased the abundance of HM species by approximately 50%. This, again, could be ascribed to differences between cell lines used in the 24-well DWP and Ambr^®^ 15 cultivation systems. L-fucose exerted the strongest decrease in HM species, by almost two-fold. Nevertheless, these modulators act nonspecifically and thereby also impact other glycan groups; as such, their use must be carefully evaluated.

As discussed previously, the modulator-induced increase in HM species is linked with an increase in afucosylation. To better analyze afucosylation and its modulators, two separate models were constructed: “afucosylation” included all glycoforms that lack core fucose, and “complex afucosylation” excluded HM glycans. This approach revealed, for example, that kifunensine only affected afucosylation by increasing HM glycoforms. Conversely, monensin increased complex afucosylation even after excluding the effect of increased HM species. This can be attributed to monensin being an ionophore. Creating pores in the membranes of the Golgi apparatus disrupts the pH balance of the organelle and prevents enzymes that are active in the latter stages of the glycosylation pathway to act on the growing glycan chain (Pande et al., 2015). By far the strongest modulator to increase complex afucosylation was 2F-peracetyl fucose, which is a strong fucosyltransferase inhibitor (Zimmermann et al., 2019). It increased complex afucosylation by up to one-fold. 2F-peracetyl fucose has specificity towards fucosyltransferases and thus does not affect other glycan groups, making it an ideal modulator for specific glycan group targeting. Conversely, L-fucose decreased complex afucosylation, albeit only by 40%. L-fucose also showed a quadratic effect on complex afucosylation as it exerted the largest effect at mid-range concentrations. Similarly to the 24-well DWP experiment, also here L-fucose decreased IgG titer, by 20% at high concentrations. This should be considered when optimizing bioprocess productivity. 2F-peracetyl fucose and L-fucose also exhibited the strongest interaction of all tested compounds. 2F-peracetyl fucose at high concentrations increased complex afucosylation by one-fold, whereas adding L-fucose at high concentrations decreased complex afucosylation back to the baseline. This indicates that 2F-peracetyl fucose is a competitive inhibitor of fucosyltransferases and can be outcompeted by a large enough concentration of L-fucose.

Other modulators that also decreased complex afucosylation were dexamethasone, lactose, N-acetylglucosamine, and uridine. Although their effects were smaller than that of L-fucose, each of them decreased complex afucosylation by approximately 20%. As such, a combination of these modulators could be used instead of L-fucose when decreased complex afucosylation is needed without a strong negative effect on antibody titer.

Same as in the 24-well DWP experiment, the strongest increases in galactosylated species were caused by galactose and Ex-Cell GAL+. Galactose had a stronger effect, as it increased galactosylation by around 1.5-fold, compared to the approximately 1-fold increase by Ex-Cell GAL+. Positive interactions between manganese, uridine, and galactose were expected, as they were reported in multiple studies (Gramer et al., 2011; Kildegaard et al., 2016; Karst et al., 2017; Ehret et al., 2018). However, the terminal galactosylation model did not detect any significant interactions between these three factors. Additionally, manganese on its own had a weak effect, increasing galactosylation by 16% at the highest concentration. Manganese acts as a co-factor for galactosyltransferases during galactosylation (Ramakrishnan and Qasba, 2001). A possible explanation for this lack of efficacy is that the cell growth media used in our experiment already contains a high enough concentration of manganese for efficient enzyme function. Uridine had a better efficacy than manganese, increasing galactosylation by 40% at high concentrations. However, it also had no significant positive interaction with either manganese or galactose. It did, however, decrease titer by 14% at high concentrations.

The model revealed interesting interactions between galactosylation modulators, namely between galactose and Ex-Cell GAL+. First, the effect curves of both modulators in the prediction profiler in Figures 3, 4 have an almost identical shape. Inspecting the model (Supplementary Table S3) for terminal galactosylation revealed an interaction between these two modulators. By increasing the concentration of one modulator in the prediction profiler, the steepness of the prediction curve for the other modulator decreases accordingly. This means that increasing the content of one of them decreases the efficacy of the other. The composition of Ex-Cell GAL+ is not known as it is a proprietary chemical mixture by Sigma-Aldrich. A possible explanation for the fact that the efficacy of galactose is decreased when applying Ex-Cell GAL + concurrently is that Ex-Cell GAL + contains galactose.

Only three modulators decreased the abundance of galactosylated species in the Ambr^®^ 15 experiment: monensin, 1-deoxynojirimycin, and N-acetylmannosamine. They all had relatively weak effects, each individually decreasing galactosylation by approximately 10%. Interestingly, monensin did not decrease galactosylation as much as other “mature” glycan groups, indicating that galactosyltransferases are more resistant to perturbations in Golgi apparatus pH levels.

As sialylated glycopeptides are the most “mature” subgroup, it is not surprising that they are the most affected by modulators acting on the glycan species that are synthesized earlier in the glycosylation pathway. The strongest positive modulators were Ex-Cell GAL+ and galactose, increasing sialylation by 1.3-fold and 0.7-fold, respectively. It is possible that Ex-Cell GAL+ is a more effective positive modulator than galactose because it contains additional components that facilitate sialylation better than galactose alone. A positive interaction was observed between galactose and dexamethasone. Dexamethasone at high concentrations very weakly increased sialylation. When combined with galactose at high concentrations, they acted synergistically and increased sialylation by one-fold. Other modulators that increased sialylation, albeit to a lesser degree, were uridine, N-acetylglucosamine, mannose, L-fucose, kifunensine, and N-acetylmannosamine. Unexpectedly, N-acetylmannosamine did not increase sialylation strongly. This may be due to its low cell membrane permeability, as cells lack a specific transporter for it. Better results have been achieved by using acetylated or butyrated N-acetylmannosamine derivatives that can passively diffuse through cell membranes with higher efficiency (Almaraz et al., 2012).

The modulator that most strongly decreased sialylation was monensin. This is due to the creation of pores in the Golgi apparatus, which alters pH and disrupts sialyltransferase activity (Müthing et al., 2003), as previously discussed for fucosylation.

One of the most important aspects of recombinant antibody production is productivity, which is why a model for the effects of the investigated modulators on antibody titer was also constructed. No strong negative effects on titer were expected, as the appropriate concentration ranges were already determined using the data from the 24-well DWP screening. Nevertheless, some modulators negatively affected titer by 10%–20%, including L-fucose, uridine, Ex-Cell GAL+, and 1-deoxynojirimycin. When applied individually, these titer reductions are not problematic, especially considering the positive effects of the modulators on the quality attributes of the produced antibodies. However, care should be taken when using these modulators together, as applying all four of them decreases titer by 66%, which is not negligible.

The model indicates only three modulators that positively affect titer: manganese, monensin, and galactose. Each of them increased titer by less than 10%, and when combined, they increased titer by 25%. However, monensin and galactose exert opposite and strong effects on HM and sialylated glycan species, which complicates their combined use, should a specific quality profile be desired.

Two factors that have not been discussed yet are the categorical factor “Cell line” and blocking factor “Block”. Some variability in measured titer depended on “Block” (Figure 4). This is due to the combination of many variable factors, including seeding cell densities, fluctuations in temperature, different Ambr^®^ 15 systems, and culture stations.

The “Cell line” factor had a significant effect in all models, except for afucosylation and complex afucosylation. The two different IgG molecules produced by the cell lines had similar afucosylation levels but differed in the proportion of other glycoforms and in productivity. Therefore, the significance of this factor is not surprising. Most investigated modulators exerted similar effects in both cell lines (e.g., a modulator increased a glycoform group in both IgG-B- and IgG-C-producing cells); however, the strength of the effects varied. This can be attributed to differences in cell metabolism and enzyme expression between the two cell lines. The only modulator that exhibited opposite effects in the two cell lines was lactose. Lactose increased the amount of HM glycans (by 40%) in IgG-B-producing cells but decreased HM glycoforms (by 37%) in IgG-C-producing cells.

## 4 Conclusion

During the production of monoclonal antibodies, N-glycosylation plays a crucial role as a post-translational modification that significantly influences the properties and effector functions of proteins. Consequently, it is considered a common critical quality attribute that necessitates careful control during the development of new therapeutic products. Traditionally, the manipulation of N-glycosylation involved genetic modification, which, although effective and precise, proved to be time-consuming and resource-intensive. Therefore, recent efforts within the pharmaceutical industry have focused on exploring the impact of small-molecule modulators on the glycosylation profiles of therapeutic proteins.

This study comprehensively investigated the effects and interactions of a wide array of glycosylation modulators using different bioprocess setups and cell lines that produce monoclonal IgG antibodies. A multi-tiered approach was used to determine the effective concentration ranges of various modulators. Initially, 24-well DWP screening was performed to identify potential modulators, which were subsequently subjected to a DoE approach for further investigation. The results obtained from the designed experiment enabled the construction of models that provided insights into concentration-dependent interactions between modulators, highlighting both synergistic and antagonistic effects on distinct glycoform groups. Notably, enzyme inhibitors, such as kifunensine, 2F-peracetyl fucose, and 1-deoxynojirimycin, exhibited the most pronounced effects. These inhibitors specifically targeted enzymes involved in the synthesis of certain glycoform groups (mannosidases and fucosyltransferases), resulting in significant alterations. Conversely, other modulators displayed less specificity, exerting broader effects on various glycoforms. These modulators primarily belonged to a group of metabolic precursors, including mannose, galactose, L-fucose, uridine, N-acetylmannosamine, and N-acetylglucosamine. Furthermore, the study compared the effects of the modulators across different cell lines that produce IgG antibodies. This revealed differences in the modulation of glycoforms, particularly concerning metabolic precursors. By contrast, enzyme inhibitors displayed consistent effects regardless of the cell line used. These findings highlight the importance of considering cell line variations and selecting appropriate modulators when implementing small-molecule modulators in bioprocesses.

Moreover, it is crucial to note that certain chemicals, including L-fucose, uridine, and 1-deoxynojirimycin, can have detrimental effects on cell productivity. Thus, careful evaluation is necessary before their utilization. Care should also be taken when using non-standard sugars such as D-arabinose, as they can incorporate into glycan structures, which is a potential safety concern. The data and models presented in this study provide valuable guidance for bioprocess scientists aiming to enhance the glycosylation profiles of their production processes. Although the effects of the examined modulators on glycosylation have been extensively characterized, their effects on other critical quality attributes, such as charge variants or aggregates, have not been investigated. Therefore, further research is warranted to comprehensively explore these aspects.

In conclusion, this study sheds new light on the effects and interactions of various small-molecule modulators on the glycosylation profiles of monoclonal antibodies. Its findings underscore the significance of controlling N-glycosylation as a critical quality attribute during the development of therapeutic products. By utilizing the provided data and models, researchers can enhance their understanding and optimize glycosylation profiles, ultimately contributing to faster and more efficient development of therapeutic proteins.

## Data Availability

The original contributions presented in the study are included in the article/Supplementary Material, further inquiries can be directed to the corresponding author.
