# Supplementary material for: Modulating antibody N-glycosylation through feed additives using a multi-tiered approach
Source: Front Bioeng Biotechnol. 2024 Aug 26;12:1448925. doi: 10.3389/fbioe.2024.1448925 (PMC11381414; doi:10.3389/fbioe.2024.1448925)
Supplement: Supplementary file 1 [file DataSheet1.zip › Supplementary Material.docx]

Supplementary Material

Modulating antibody N-glycosylation through feed additives using a multi-tiered approach

Jaka Kranjc^1^, Lovro Kramer^2^, Miha Mikelj^3^, Marko Anderluh^4†^, Anja Pišlar^5†^, Matjaž Brinc*^6†^

^1^Institute for Pharmacy, Faculty of Pharmacy, University of Ljubljana, Ljubljana, Slovenia

^2^Cell Line Engineering and Characterization, Technical Research & Development, Novartis Pharmaceutical Manufacturing LLC, Mengeš, Slovenia

^3^Process Analytical Science, Technical Research & Development, Novartis Pharmaceutical Manufacturing LLC, Mengeš, Slovenia

^4^Department of Pharmaceutical Chemistry, Faculty of Pharmacy, University of Ljubljana, Ljubljana, Slovenia

^5^Department of Pharmaceutical Biology, Faculty of Pharmacy, University of Ljubljana, Ljubljana, Slovenia

^6^Process development, Technical Research & Development, Novartis Pharmaceutical Manufacturing LLC, Mengeš, Slovenia

*** Correspondence:**Matjaž Brinc
[matjaz.brinc@novartis.com](mailto:matjaz.brinc@novartis.com)

^†^These authors contributed equally to this work and share senior authorship

# Supplementary Table Captions

Supplementary Table 1: Factors used for the design of experiment performed in Ambr15 and measured glycosylation patterns. Glycosylation data was normalized versus a control experiment. Each row corresponds to one bioreactor.

Supplementary Table 2: Normalized heatmap table of measured glycan structures from the 24-well deep well plate experiment. Numbers in the glycan structure columns represent fold changes compared to control. Values higher and lower than the mean are highlighted in green and red, respectively. The stronger the intensity of the color, the larger the delta from the mean.

Supplementary Table 3: Report table exported from JMP 17 containing statistical parameters for models constructed based on the glycosylation pattern data obtained from the Ambr15 design of experiment.

**
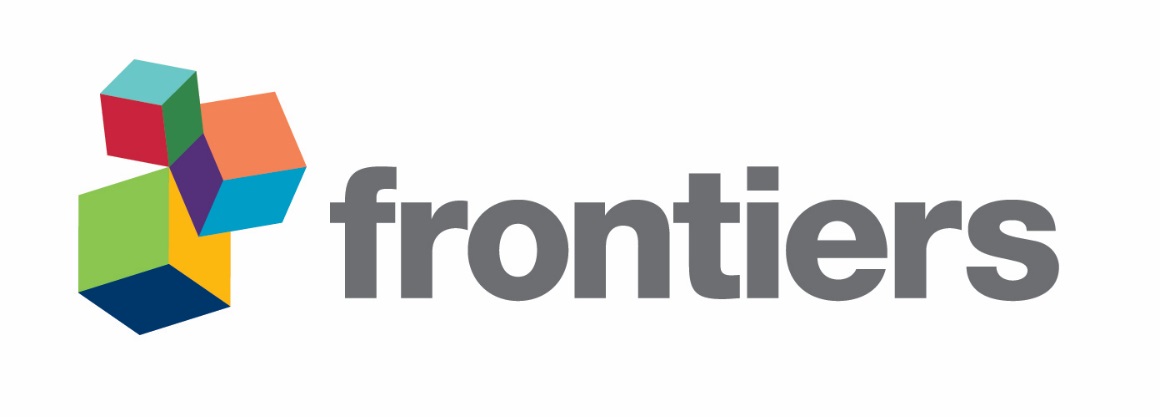
**
