# Supplementary material for: Modulating antibody N-glycosylation through feed additives using a multi-tiered approach
Source: Front Bioeng Biotechnol. 2024 Aug 26;12:1448925. doi: 10.3389/fbioe.2024.1448925 (PMC11381414; doi:10.3389/fbioe.2024.1448925)
Supplement: Supplementary file 1 [file DataSheet1.zip › Supplementary Table 3.html]

Ambr15 DoE za članek zamaskiran in samo podatki za objavo - Fit Group
